# A Framework for Leveraging “Big Data” to Advance Epidemiology and Improve Quality: Design of the VA Colonoscopy Collaborative

**DOI:** 10.5334/egems.198

**Published:** 2018-04-13

**Authors:** Samir Gupta, Lin Liu, Olga V. Patterson, Ashley Earles, Ranier Bustamante, Andrew J. Gawron, William K. Thompson, William Scuba, Daniel Denhalter, M. Elena Martinez, Karen Messer, Deborah A. Fisher, Sameer D. Saini, Scott L. DuVall, Wendy W. Chapman, Mary A. Whooley, Tonya Kaltenbach

**Affiliations:** 1 VA San Diego Healthcare System, US; 2 University of California, San Diego, US; 3 VA Salt Lake City Health Care System, US; 4 Northwestern University, US; 5 University of Utah, US; 6 Durham VA Medical Center, US; 7 VA Ann Arbor Healthcare System, US; 8 San Francisco VA Medical Center, US

**Keywords:** big data, electronic health records, epidemiology, quality improvement, veterans

## Abstract

**Objective:**

To describe a framework for leveraging big data for research and quality improvement purposes and demonstrate implementation of the framework for design of the Department of Veterans Affairs (VA) Colonoscopy Collaborative.

**Methods:**

We propose that research utilizing large-scale electronic health records (EHRs) can be approached in a 4 step framework: 1) Identify data sources required to answer research question; 2) Determine whether variables are available as structured or free-text data; 3) Utilize a rigorous approach to refine variables and assess data quality; 4) Create the analytic dataset and perform analyses. We describe implementation of the framework as part of the VA Colonoscopy Collaborative, which aims to leverage big data to 1) prospectively measure and report colonoscopy quality and 2) develop and validate a risk prediction model for colorectal cancer (CRC) and high-risk polyps.

**Results:**

Examples of implementation of the 4 step framework are provided. To date, we have identified 2,337,171 Veterans who have undergone colonoscopy between 1999 and 2014. Median age was 62 years, and 4.6 percent (n = 106,860) were female. We estimated that 2.6 percent (n = 60,517) had CRC diagnosed at baseline. An additional 1 percent (n = 24,483) had a new ICD-9 code-based diagnosis of CRC on follow up.

**Conclusion:**

We hope our framework may contribute to the dialogue on best practices to ensure high quality epidemiologic and quality improvement work. As a result of implementation of the framework, the VA Colonoscopy Collaborative holds great promise for 1) quantifying and providing novel understandings of colonoscopy outcomes, and 2) building a robust approach for nationwide VA colonoscopy quality reporting.

## Introduction

Worldwide adoption of electronic health records (EHRs) holds great promise for ushering in a new era of epidemiology, in which “big data” can be leveraged to advance our understanding of epidemiology of rare diseases and inform quality improvement initiatives. While the promise is real, extracting research-grade data from EHRs is a challenge[1]. First, most clinical encounters are captured as free-text clinical notes, which in raw form, are not suitable for research purposes. Second, even when structured data are available, challenges such as data entry errors and misclassification of coding remain. For example, an International Classification of Diseases, 9^th^ Revision (ICD-9) diagnoses code for colorectal cancer (CRC) might have been entered for a patient undergoing colonoscopy based on clinical suspicion for cancer, which was ultimately ruled out by normal colonoscopy. In other instances, an ICD-9 diagnosis code may not have been entered for a given healthcare encounter, reducing the sensitivity of an ICD-9 code-based approach for identifying diagnoses.

**Figure 1:**
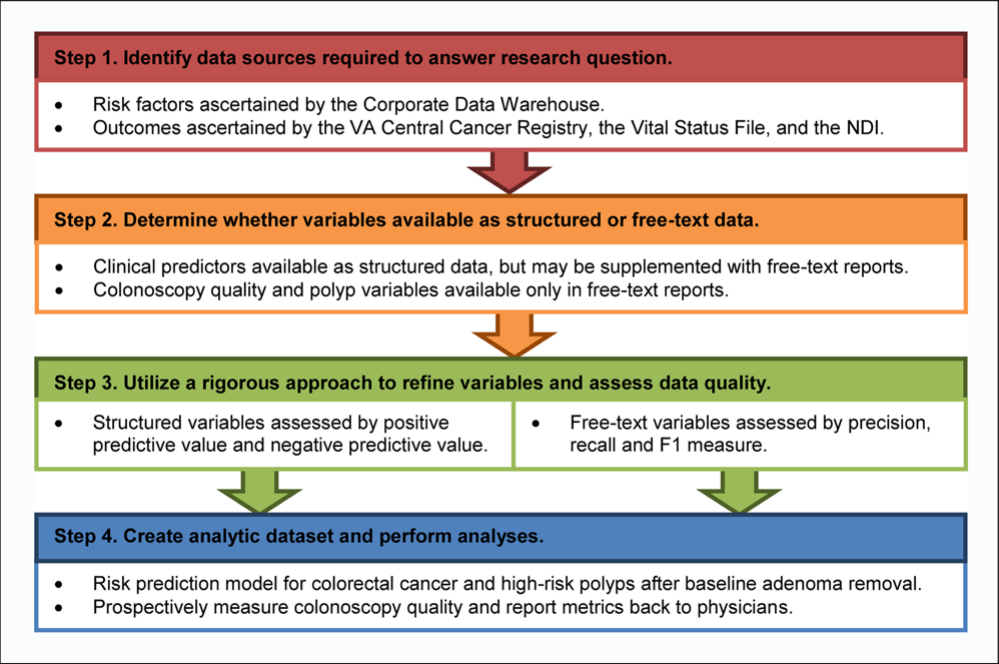
**Framework for leveraging “big data” for research and quality improvement purposes.** Examples of implementation for each step proposed for the VA Colonoscopy Collaborative are provided. VA, Department of Veterans Affairs; NDI, National Death Index.

A major challenge to research utilizing large-scale EHRs is the curation and validation of data of interest. Intelligent application of novel informatics tools, coupled with thoughtful adherence to basic epidemiologic principles for assessing misclassification may address these limitations. Indeed, a rigorous approach must be taken to validate EHR data because failing to do so may result in biased epidemiological research and incomplete quality improvement efforts. We herein describe a framework for leveraging big data for research and quality improvement purposes and then demonstrate implementation of the framework for design of the Department of Veterans Affairs (VA) Colonoscopy Collaborative (Figure 1).

## Methods

### Rationale for use case

CRC is the second leading cause of cancer death in the United States (US) [2]. Incidence and mortality can be reduced by detection and removal of potentially precancerous polyps and CRC. In the US, colonoscopy is the most commonly used test to prevent and diagnose CRC [3]. While exposure to colonoscopy has been associated with reduced CRC incidence and mortality, several challenges remain. First, outcomes among individuals with reportedly normal colonoscopy are highly variable. Observational studies have demonstrated up to 12-fold variation in CRC incidence and mortality after normal colonoscopy, likely due to variation in ability to detect polyps and cancer across physicians [4,5]. To optimize colonoscopy quality, several quality metrics have been recommended for routine monitoring, including adenoma detection rate, adequate bowel preparation, and complete extent of examination. Adenoma detection rate, defined as the proportion of screening colonoscopies that detect at least one histologically-confirmed adenoma or CRC, is of particular interest, as variation in adenoma detection rate has been linked to both risk for post-colonoscopy CRC incidence and mortality [4,5]. Despite consensus recommendations for quality measurement, impact of large-scale implementation and monitoring of recommended colonoscopy metrics and ability to predict outcomes remains uncertain [6].

Variable outcomes among individuals who have had polyps identified and removed is a second challenge. For example, one long-term follow-up study observed persistently elevated CRC mortality among patients who had “high-risk polyps” (defined as an adenoma with high grade dysplasia, tubulovillous or villous histology, and/or size greater than 1 cm) removed at baseline [4]. However, evidence to support whether surveillance colonoscopy impacts CRC incidence and mortality (beyond the initial polypectomy) is quite limited. National guidelines, such as those by the US Multi-Society Task Force on Colorectal Cancer, recommend routine surveillance for all patients with adenoma removal, with timing guided by the number, size, and histology of baseline findings [7]. However, these recommendations are based on studies that used CRC and high-risk polyps (also defined as metachronous advanced neoplasia) as the endpoint, and most data used to guide recommendations were from research studies with interventions to reduce polyp recurrence. Epidemiologic research in this area is logistically challenging, because polyps must be characterized with respect to size, number, and histology, based on information that comes from free-text colonoscopy and pathology reports. In particular, histology is a critical variable, since many polyps are benign (non-neoplastic).

**Figure 2:**
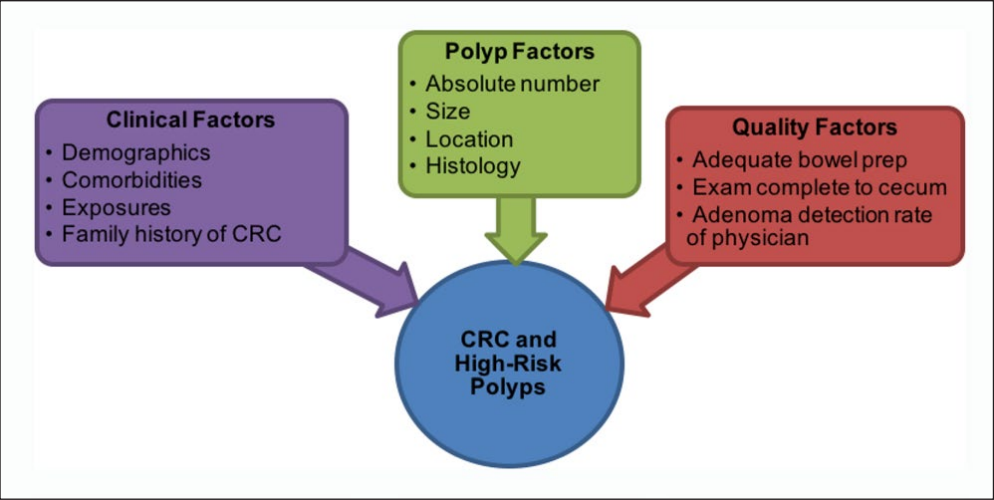
**Risk Factors for CRC and High-Risk Polyps after Baseline Adenoma Removal.** The VA Merit Review-supported cohort study will consider the potential influence of established and suspected risk factors for CRC and high-risk polyps. CRC, colorectal cancer.

To realize the full promise of colonoscopy for reducing CRC incidence and mortality, extensive epidemiologic research and quality improvement initiatives are required to understand and mitigate the impact of factors associated with CRC and high-risk polyps after baseline adenoma removal. The VA houses one of the largest repositories of data collected and maintained as part of usual healthcare. As such, this setting provides a unique opportunity to advance our understanding of epidemiology and improve quality across the VA healthcare system. Accordingly, we have initiated the VA Colonoscopy Collaborative, funded through two distinct mechanisms including 1) a VA Quality Enhancement Research Initiative (QUERI), which will prospectively measure colonoscopy quality and report these metrics back to physicians, and 2) a VA Merit Review-supported cohort study, which will develop and validate a risk prediction model for CRC and high-risk polyps after baseline adenoma removal. The cohort study will consider the potential influence of established and suspected risk factors for CRC and high-risk polyps (Figure 2).

### Overview of approach to leverage big data for research and quality improvement purposes

We herein describe a framework for leveraging large-scale EHR data to conduct epidemiological and quality improvement research and then demonstrate implementation of the framework for design of the VA Colonoscopy Collaborative (Figure 1). Below we review in detail the rationale for each step, and provide an example relevant to our project that demonstrates how we implemented the approach to design the VA Colonoscopy Collaborative.

## Step 1. Identify data sources required to answer research question

Rationale: In the new era of availability of large-scale EHRs for research and quality improvement, careful attention must be taken to identify the optimal data sources for answering research questions. For example, resources that capture larger, more representative populations should be chosen over more restrictive ones, and efforts should be taken to identify EHRs with routinely captured data most critical for the research question. Specifically, the VA’s EHR has the advantage of potential linkage to National Death Index data, which allows ascertainment of cause-specific mortality, a critical variable for many epidemiologic studies. Similarly, EHRs with opportunities for more rigorous ascertainment of diagnoses and/or outcomes should be chosen over those with more limited data. For example, some EHRs only allow for identification of patients with iron deficiency anemia (a risk factor for CRC diagnosis) through ICD-9 codes, while others offer direct access to structured lab values for much more rigorous ascertainment of iron status if measured as part of usual care. Decisions on which data sources to access for an epidemiologic or quality improvement study should not simply be based on sample volume, as sample size will not be an immunization against bias.

**Figure 3:**
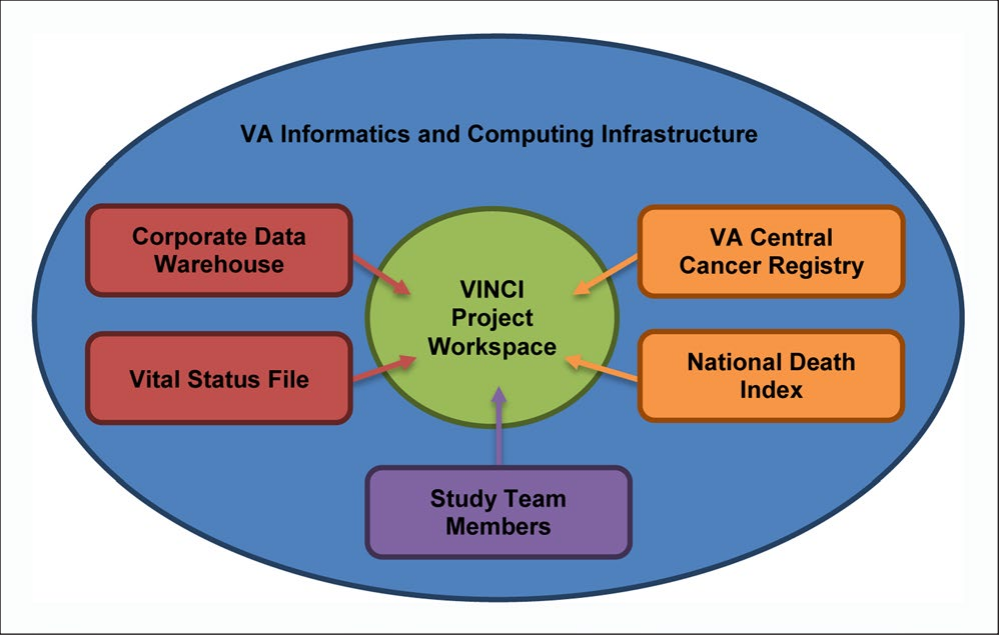
**Data Sources Available within VINCI.** Data sources potentially accessible through VINCI include data created and maintained as usual healthcare, as well as external data sources that can be uploaded to VINCI. VA, Department of Veterans Affairs, VINCI, VA Informatics and Computing Infrastructure.

Example: The VA houses one of the world’s largest data repositories and make possible efforts such as the VA Colonoscopy Collaborative. For research, as well as quality improvement initiatives, VA data can be accessed through the VA Informatics and Computing Infrastructure (VINCI), a secure, central analytic platform for performing research and supporting clinical operations activities [8]. Data sources potentially accessible through VINCI include data created and maintained as usual healthcare (e.g., Corporate Data Warehouse), as well as external data sources (e.g., VA Central Cancer Registry) that can be uploaded to VINCI. Accessible data include administrative billing codes (e.g., ICD-9 diagnosis codes), structured laboratory data, medications, and free-text reports for a wide array of clinical services, such as outpatient clinic visits, procedures, surgeries and pathology reports. Approved projects are provided a VINCI project workspace and all Institutional Review Board-approved study team members are granted access, greatly facilitating collaborative research, code sharing, and the ability to collaborate across multiple sites. For this initiative, a VINCI project workspace has been created that includes access to all of the data sources indicated in Figure 3.

## Step 2. Determine whether variables available as structured or free-text data

Rationale: Once the best data sources to answer the research question have been identified, careful consideration of each variable required for ascertaining inclusion and exclusion criteria, predictors, exposures, and key outcomes and determination of whether each variable is available as structured or free-text data is required. Recognizing that sophisticated techniques such as natural language processing (NLP) take significant resources for development and validation, as well as for maintenance if ongoing ascertainment is required, whenever possible, preference should be for identification and validation of structured data created as part of usual care for variable ascertainment. However, variable concepts for which adequate structured data are not available may be considered for NLP algorithm development. Pre-specifying variables that will be ascertained using structured vs. free-text data allows for clear assignment of tasks for the study team, with one or more members focused on optimizing structured data, and the other team members focused on unlocking the potential of free-text data using advanced informatics approaches such as NLP.

Example: As part of work on the VA Colonoscopy Collaborative, we have identified several clinical predictors (e.g., demographics, comorbidities, exposures) required for the proposed work were available as structured data. However, a number of key predictors and outcomes required for the proposed work were only available in free-text form. For example, polyp characteristics (e.g., number, size, location, and histology) and colonoscopy quality (e.g., adequate bowel preparation and completeness of exam) were only available in colonoscopy and pathology reports and were selected for development of NLP algorithms for variable extraction from free-text reports. A list of target study-related variables and their availability as structured vs. free-text data are provided in Appendix A. All curated variables will be stored within our VINCI project workspace. A data dictionary detailing the variable name, definition, and data source has been created and will be updated regularly (summarized in Appendix B).

## Step 3. Utilize a rigorous approach to refine variables and assess data quality

Rationale: While large-scale EHR data offer the opportunity to answer questions that require large sample size and/or finding rare outcomes with precise estimates, misuse can also result in magnification of systematic bias. For example, use of pooled EHR data from 50 clinical care sites might offer sufficient power to answer an array of research questions, but if an outcome variable is systematically under-ascertained from patients from a few, but not all clinical care sites contributing data, biased estimates will result. Similarly, understanding the strengths and weaknesses of an NLP-derived outcome variable allows for more careful selection of final variables for analyses, and more nuanced interpretation of study outcomes. For example, an NLP derived variable with very high precision, but suboptimal recall could be used to estimate the minimum rate of an outcome, but would not be represented as the true population rate, and would need to be supplemented with additional outcome ascertainment strategies.

Example: We implemented similar techniques to curate both structured and free-text data. Below we outline our plans to develop and validate structured and free-text variables separately.

*Structured Variables:* To date, we have curated a number of clinical predictors required for the proposed work. We have also implemented a number of statistical techniques to assess data quality, including conformance, completeness and plausibility [9]. For categorical variables (e.g., race/ethnicity), extent of missing data were evaluated. For continuous variables (e.g., height and weight), descriptive statistics were utilized to assess distribution and extent of missing data. Particular attention was paid to logic checking and data cleaning. For some variables, algorithms to identify certain diagnoses using administrative data have undergone prior development and validation, and were implemented directly. For example, published approaches to identification of diabetes, smoking and body mass index among Veterans exist, and have been implemented for our work [10,11,12].

For other variables, limited or no prior work had been done, requiring rigorous development of strategies for structured data curation and validation. Our general approach has been to create an initial strategy to identify potential cases and controls for a predictor or outcome of interest, drawing from one or more structured data fields. Performance of the strategy was then compared against manual chart review. Positive predictive value (PPV) and negative predictive value (NPV) were estimated by taking a random sample of potential cases and potential controls, and reviewing charts to confirm diagnoses and note errors. PPV and NPV were then combined with estimated prevalence to calculate sensitivity and specificity. Our process for structured variable development and validation is described in detail in Appendix C. Notably, we estimated the sample size needed for manual chart review based on a pre-specified target threshold for accuracy measures (PPV and NPV) and outlined a stepwise validation process. We believe that employing this approach for developing and validating structured variables will enhance efficiency and reduce potential for biased reporting.

When multiple strategies for variable ascertainment using structured data are under consideration, additional steps beyond assessment of PPV and NPV for each individual strategy are required, including comparison of agreement across strategies, as well as review of discordant cases. We have implemented this approach for comparing strengths and weakness of three CRC diagnosis strategies available through VA data including: administrative claims data consisting of ICD-9 diagnosis codes for CRC; the VA Central Cancer Registry; and a novel resource that has not been previously validated consisting of cases abstracted by local cancer registrars (the Oncology Domain). Utilizing the three-pronged approach of assessing PPV and NPV for candidate CRC cases and non-CRC controls for each strategy, comparing agreement across strategies, and review of a sample of discordant cases was highly informative. We found cancer registry and Oncology Domain diagnoses both had high PPV and agreement with one another, and that while ICD-9 based diagnoses had low PPV and poor agreement with the other two sources, that a substantial number of ICD-9 flagged, but cancer registry unflagged, as well as Oncology Domain unflagged cases had true CRC [13]. As such, the multi-pronged approach allowed us to fully assess the strengths and weaknesses of the three approaches for cancer ascertainment within our large dataset.

**Figure 4:**
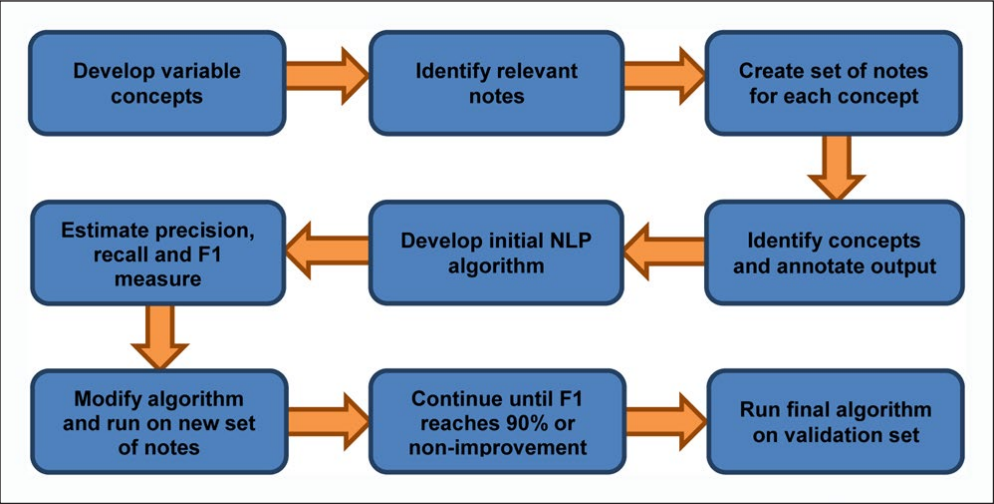
**Workflow for NLP Algorithm Development and Validation**.

*Free-Text Variables:* We have initiated development and validation of NLP algorithms to extract data only available in free-text colonoscopy and pathology reports (Figure 4).

First, we developed explicit, unique concepts for each variable of interest, by defining the concept, justifying its importance, and identifying potential ways the concept might be expressed in candidate reports (see Appendix D for the variable concept sheet for bowel preparation). Next, we created an initial set of annotated documents for each variable concept, which was created by stratified random sampling candidate source reports to ensure that all available VA facilities were represented in the initial set. Subsequently, annotators were trained to identify evidence within the reports that indicated presence/absence of each concept and annotated the output accordingly. Annotation was performed using a chart annotation tool available in the VINCI project workspace [14]. Subsequently, the annotated data and corresponding reports were used to develop NLP algorithms to extract data for each variable of interest. Once an initial algorithm for a given variable concept was finalized, it was run on another random sample of free-text reports. Accuracy of each algorithm will be assessed using standard NLP system evaluation metrics of recall, precision, and F1 measure [15]. We will iteratively continue the annotation, algorithm, re-annotation process until each algorithm reaches ≥ 90 percent F1 measure for variable identification, or a threshold of non-improvement. An independent, annotated sample of reports will be selected to provide a best estimate of each final algorithm’s performance. Finally, the algorithms, once finalized, will be applied to the entire corpus of candidate reports to output NLP-related variables for our quality improvement and epidemiologic analyses.

## Step 4. Create analytic dataset and perform analyses

Rationale: Once all variables have been finalized, the final step is to create an analytic dataset and perform analyses specific to relevant research questions, utilizing best practices. Here, the main concern specific to use of large-scale data is the potential for systematic bias to have a magnified impact. Care must be taken to carefully assess missingness and other data patterns not just across patients, but at all levels that could influence data availability, such as at the provider, clinic, and even health system level. When interpreting results, at least three best practices should be considered. First, when considering the clinical significance of findings, absolute, as well as relative risks should be carefully examined, as large sample sizes might allow for finding significant differences in relative risks that are too rare to be clinically meaningful. Similarly, emphasis should be placed on the clinical, over the statistical significance of results, as large sample sizes will often result in statistically significant results at traditional thresholds (such as the commonly used p < 0.05 threshold) that may not be relevant. Third, alternate explanations for results, with particular attention to errors introduced by the data curation processes implemented, as well as source of data must be considered carefully.

Example: Below we outline the analysis plans for the epidemiologic study and quality improvement initiative separately.

*Development of Risk Prediction Models:* To improve colonoscopy surveillance, we will develop and validate a risk prediction model for CRC and high-risk polyps among individuals who had a baseline adenoma removal. The study sample will be all Veterans with index colonoscopy where an adenomatous polyp or sessile serrated adenoma/polyp was removed. We will exclude individuals with CRC or inflammatory bowel disease prior to or at time of index colonoscopy. The primary outcome will be subsequent CRC or high-risk polyps. Predictors of interest will include clinical factors (e.g., smoking), polyp factors (e.g., size) and quality factors (e.g., adenoma detection rate). Our planned process for model development and validation is described in detail in Appendix E. Notably, we will set two cut-points for the prediction model that improve sensitivity and specificity over the current US Multi-Society Task Force on Colorectal Cancer guidelines based on model development within the training set, and use these same cut-points for model validation within the independent validation set. Absolute rates of advanced neoplasia on follow up will be contrasted based on guideline-based, as well as model-based risk stratification, with emphasis on evaluating clinically significant improvements in sensitivity and specificity (e.g., ≥ 10 percent differences) for the model over guideline-based risk stratification.

*Assessment of Colonoscopy Quality Metrics:* To improve colonoscopy quality, we will measure and report quality metrics to physicians. The three initial quality metrics are 1) adenoma detection rate; 2) adequate bowel preparation, and 3) complete extent of examination. The study sample will consist of Veterans with a screening colonoscopy performed within the VA healthcare system. Colonoscopies will be identified and quality metrics will be characterized on a “real-time” basis. This will involve working with operational partners to host EHR data in a secure environment. Colonoscopy quality report cards will be generated for each VA facility and for individual providers within that facility. Steps will be taken to characterize VA sites for differences in region, patient populations, and other characteristics that might account for differences to ensure observed differences are attributable to colonoscopist performance. These report cards will also highlight recommended performance targets. Results will be characterized and tracked at the national VA system level to determine whether feedback improves performance over time. Processing of individual VA facility and provider level data will occur at least annually, and the colonoscopy quality report card will be implemented using a randomized, stepped wedged design which will greatly facilitate evaluation across VA facilities [16].

## Preliminary Results

To date, we have identified 2,337,171 Veterans with at least one colonoscopy performed within the VA healthcare system 1999–2014 and summarized baseline characteristics (Table 1). Median age was 62 years, and 4.6 percent (n = 106,860) were female. We estimate that 2.6 percent (n = 60,517) had CRC at baseline. Additionally, over 26 percent (n = 624,262) had at least 1 follow-up colonoscopy, and 1 percent (n = 24,483) had a new ICD-9 code-based diagnosis of CRC on follow up. The high absolute number of Veterans with potential CRC after colonoscopy emphasizes the great potential of the cohort for analyzing CRC alone as well as in combination with high-risk polyps.

**Table 1:** Baseline Characteristics of Veterans with Colonoscopy 1999–2014.

Variables	*N* = 2,337,171	

Age, years, median (Q1–Q3)	62	(55–69)
Sex, n (%)		
Male	2,230,311	–95.4
Female	106,860	–4.6
Race/ethnicity, n (%)		
Non-Hispanic white	1,615,606	–69.1
Non-Hispanic black	359,981	–15.4
Hispanic	98,884	–4.2
Asian	26,399	–1.1
American Indian	11,661	–0.5
Multiracial	39,553	–1.7
Unknown	185,087	–7.9
Body mass index, kg/m^2^, median (Q1–Q3)	28.9	(25.7–32.7)
Diabetes, n (%)	534,086	–22.9
Medications, n (%)		
Non-steroidal anti-inflammatory drugs (NSAIDs)	1,019,098	–43.6
Statins	1,038,017	–44.4
Inflammatory bowel disease, n (%)	21,468	–0.9
Colorectal cancer* (CRC), n (%)		
CRC at baseline	60,517	–2.6
CRC on follow up	24,483	–1
Pathology report within 30 days of baseline colonoscopy, n (%)	586,183	–25.1
Follow-up colonoscopy, n (%)	624,262	–26.7

* CRC based off International Classification of Diseases, 9^th^ Revision diagnosis codes

Progress in assessment of variation of adenoma detection across VA sites has been made. For example, application of a search strategy for mention of adenomas within free-text pathology reports among 105,553 individuals who had colonoscopy at one of 96 VA sites in the year 2014 averages 39.6 percent, but ranges from 8.7 percent to 64.3 percent across sites, consistent with clinically significant variation in quality of colonoscopies performed.

## Discussion

Extracting research-grade data from EHRs is a challenge. We propose a framework for conducting research and quality improvement projects utilizing large-scale EHRs that may optimize efficient production of interpretable results. Our preliminary data suggests that these approaches have great potential for enabling large-scale epidemiologic research and quality improvement initiatives. As is the case with use of data created as part of healthcare subsequently used for research and quality improvement, limitations are expected. For example, reliable predictor variables can only be developed for conditions that are documented consistently in the EHR. Family history of cancer is often under-reported, and may be under-represented in our analytic cohort [17]. As such, if we do not find an association between family history and important outcomes, a type II error due to under-ascertainment of family history must be considered. Second, as was shown to be the case for CRC case identification, multiple ascertainment strategies may need to be utilized for the same variable, depending on the analysis. As a result of implementing multiple strategies, we can determine best practices for ascertainment, depending on the analysis at hand. Specifically, for measuring incident CRC, we may consider primary ascertainment based on registry-based CRC diagnosis, but also conduct a sensitivity analysis in which both cancer registry and ICD-9 based approaches are used to define incident CRC. If a case-control analysis is conducted, CRC cases may be defined based on the ascertainment strategy with the highest specificity, to avoid incorrect attribution that might bias towards null findings. Additional variables may require careful testing and evaluation of multiple ascertainment strategies to optimize our work. A third potential limitation of our work is generalizability to other healthcare systems. However, there is some evidence to suggest that algorithms derived from EHRs can be successfully adapted to other healthcare systems [18].

## Conclusion

Several outcomes are anticipated as a result of the VA Colonoscopy Collaborative. We hope the collaborative is a model for bringing together researchers and operational partners to leverage big data for research and quality improvement purposes. Due to large sample size and curation of a broad array of structured as well free-text data from colonoscopy and pathology reports, the collaborative holds promise for 1) quantifying and providing novel understandings of colonoscopy outcomes, and 2) building a robust approach for nationwide VA colonoscopy quality reporting. By sharing our methods, we hope to contribute to the dialogue on best practices for leveraging big data for large-scale epidemiologic studies and quality improvement efforts. Whereas previously many studies have not rigorously defined and validated predictor and outcome variables with consistency, we believe a new era is emerging in which the granularity of data available demands such validation prior to conducting extensive analyses. In the extreme, without such validation work, use of large-scale EHRs has the potential to greatly increase the chances of false positive (type I) or false negative (type II) errors with respect to associations between predictors and key outcomes, and detract from the great potential for big data to advance epidemiology and improve quality.

## Additional Files

10.5334/egems.198.s1 Appendix B. Sample of Variables in Data Dictionary.

10.5334/egems.198.s1 Appendix C. Structured Variable Development and Validation Process.

10.5334/egems.198.s1 Appendix D. Variable Concept Sheet for Bowel Preparation.

10.5334/egems.198.s1 Appendix E. Risk Prediction Model Development and Validation.
