# Supplementary material for: A Framework for Leveraging “Big Data” to Advance Epidemiology and Improve Quality: Design of the VA Colonoscopy Collaborative
Source: EGEMS (Wash DC). 2018 Apr 13;6(1):4. doi: 10.5334/egems.198 (PMC5983017; doi:10.5334/egems.198)
Supplement: Appendix B. — Sample of Variables in Data Dictionary. [file egems-6-1-198-s1.pdf]

Appendix A. Variables of Interest for the VA Colonoscopy Collaborative

Variable	Data Source	Approach for Missing Data*
**Variables based on structured data**		
Age	CDW SPatient	Impute
Sex	CDW SPatient	Impute
Race/ethnicity	CDW SPatient	Impute
Body mass index (BMI)	CDW VitalSign	Impute
Statins	CDW Phamacy†	Assume non-exposure
Nonsteroidal anti-inflammatory drugs	CDW Phamacy†	Assume non-exposure
Aspirin	CDW Phamacy†	Assume non-exposure
Diabetes	CDW Diagnoses	Impute
Smoking	CDW HealthFactor	Impute
Colorectal cancer (CRC)	VACCR‡	Assume not present because rare outcome
**Variables based on free-text data**		
Colonoscopist	Colonoscopy report	Exclude from analysis
Procedure indication	Colonoscopy report	Impute
Bowel preparation	Colonoscopy report	Impute; will vary values in sensitivity analyses
Extent of exam	Colonoscopy report	Assume cecum not reached, exclude from analysis
Adenoma§	Pathology report	Assume no adenoma
Advanced adenoma§	Pathology report	Assume absent
Polyp histology§	Pathology report	Impute
Polyp dysplasia§	Pathology report	Impute
Polyp villousity§	Pathology report	Impute
Polyp location§	Both reports	Impute
Polyp size§	Colonoscopy report	Impute
Polyp shape§	Colonoscopy report	Impute
Resection method§	Colonoscopy report	Impute
Retrieval method§	Colonoscopy report	Impute
Family history of CRC	Colonoscopy report	Assumed absent if not documented
Adenoma detection rate	Both reports	Impute

CDW, Corporate Data Warehouse; VACCR, VA Central Cancer Registry.
* Missing data will be treated as an ‘unknown’ category or imputed by multiple imputation as appropriate.
† Will also consider Non-VA Medications
‡ Will also consider the Oncology Domain within the CDW
§ Not applicable if a given colonoscopy report does not document polypectomy

Appendix B. Sample of Variables in Data Dictionary

Variable	Definition	Data Source	Comment
Age	Age in years	CDW SPatient	Age was calculated as the difference between ProcedureDate and DateOfBirth from SPatient file. Excluded those <18 and ≥99.
Sex	0 = Male<br>1 = Female	CDW SPatient	Sex was curated from SPatient file.
Race/ethnicity	0 = White<br>1 = Black<br>2 = Hispanic<br>3 = Asian<br>4 = American Indian<br>5 = Other<br>6 = Unknown	CDW SPatient	Race/ethnicity was primarily identified from the Patsub files and supplemented with SPatient file. Ethnicity took priority over race and 'self-reported' recordings took priority.
Body mass index	(weight in pounds/(height in inches)^2) *703	CDW VitalSign	Obtained height and weight values up to one year prior to baseline colonoscopy. Height <48 or >84 inches excluded and modal height selected. Weight <70 or >700 pounds excluded and median weight selected. BMI <14 or >50 kg/m^2 excluded.

CDW, Corporate Data Warehouse.

Appendix C. Structured Variable Development and Validation Process

We have implemented a stepwise approach to estimate the sample size needed for manual chart review based on one-sided confidence lower bounds for positive predictive value (PPV) and negative predictive value (NPV). Bonferroni correction was used for multiple comparison adjustment. That is, to ensure an overall 95 percent confidence, a one-sided 97.5 percent confidence lower bound was calculated for PPV and NPV, respectively. We implemented a sensitivity analysis by considering a range of sample sizes (100-250 potential cases and 100-250 potential controls) and a range of estimated PPV/NPV (0.85-0.95) and adopted the following validation process:

1. Take a random sample of 100 putative cases and 100 putative controls for the predictor, exposure or outcome of interest. If the estimated PPV (based on 100 putative cases) and NPV (based on putative 100 controls) are 0.95 or greater, the confidence lower bounds for PPV and NPV will both be greater than 0.90 and therefore, we can claim the true PPV and NPV are greater than 0.90 with 95 percent confidence. An application of Bayes' theorem shows that with the above estimated PPV and NPV, the sensitivity and specificity will be at least 0.68 if the prevalence of the outcome is 0.10-0.90.

2. If estimated PPV or NPV in Step 1) are lower than 0.95, we will assess the source of errors, modify the algorithm to improve the PPV and NPV and manually review a random sample of 150 putative cases and 150 putative controls in this step. If estimated PPV and NPV are >0.90, the confidence lower bounds for PPV and NPV will be greater than 0.85 and we will claim the true PPV/NPV are greater than 0.85 with 95 percent confidence. With the above estimated PPV and NPV, the estimated sensitivity and specificity will be at least 0.69 if the prevalence is 0.20-0.80.

3. If estimated PPV or NPV in Step 2) are < 0.90, we will assess the source of errors and modify the algorithm again and manually review another random sample of 150 putative cases and 150 putative controls, estimate PPV, NPV, sensitivity, and specificity and the validation process is completed.

Appendix D. Variable Concept Sheet for Bowel Preparation

Importance: Bowel preparation refers to the quality with which the colon was cleansed, as observed at the time of a colonoscopy procedure. Quality of bowel preparation impacts ability of the colonoscopist to see polyps and cancers, and impacts recommendations for follow up. For example, a suboptimal bowel preparation might prompt a recommendation for an early 5 year instead of 10 year colonoscopy in a person with otherwise normal examination.

Variation: There is variation in the terminology used to define bowel preparation. Sometimes, more than one description is provided – e.g., bowel prep was excellent and adequate; OR bowel prep was good except in ascending colon, where the prep was fair.

Possible values of interest:

Variable	Output	Example
prep_adequate	1=yes, NULL=not found	adequate prep
prep_inadequate	1=yes, NULL=not found	prep quality was inadequate
prep_excellent	1=yes, NULL=not found	bowel prep was excellent
prep_good	1=yes, NULL=not found	visualization: good
prep_fair	1=yes, NULL=not found	quality of bowel prep: fair
prep_poor	1=yes, NULL=not found	poor preparation
prep_clean	1=yes, NULL=not found	entire colon was clean
prep_fairly clean	1=yes, NULL=not found	colon was fairly clean
prep_optimal	1=yes, NULL=not found	quality of bowel prep was optimal
prep_suboptimal	1=yes, NULL=not found	suboptimal prep
prep_boston	0-9; NULL=not found	Boston bowel prep score equal to 9
prep_ottawa	0-14; NULL=not found	Ottawa bowel prep score: 14
prep_unspecified	varchar(100)	none of the above, value other than above

* It is possible for the same report to have multiple values; for example, the same report might say that the prep was good and adequate, or good except for fair in the ascending colon; analytically, we will note these based on procedures that are associated with a “1” coded for more than one bowel prep variable.

Desired output:

norm_inadequateprep	norm_adequateprep	norm_unknown
Presence of any normalized inadequate criteria, including at least one of the following:<br>• prep_inadequate<br>• prep_fair<br>• prep_poor<br>• prep_fairly clean<br>• prep_suboptimal<br>• prep_boston 0 – 5<br>• prep_ottawa 4 – 14	Absence of all normalized inadequate criteria, plus at least one of the following:<br>• prep_adequate<br>• prep_excellent<br>• prep_good<br>• prep_clean<br>• prep_optimal<br>• prep_boston 6 – 9<br>• prep_ottawa 0 – 3	Bowel prep not assessable/missing

Appendix E. Risk Prediction Model Development and Validation

Model Development and Identification of Cut-Points for Risk Stratification

Study cohort will be randomly split into training and validation sets with 2:1 ratio. Model development will be conducted using the training set. Risk factors that are significantly (*P*<0.15) associated in univariate analysis will be considered as potential predictors for CRC and high-risk polyps in a multivariable logistic regression model. We will use a L1-regularized logistic regression model[1, 2] and Bayesian model averaging[3] for variable selection, which are considered superior to traditional stepwise model selection approaches.[4, 5] Discrimination and calibration of the selected models will be assessed using the Area under the Receiver Operating Characteristic Curve (AUC) and the Hosmer-Lemeshow goodness-of-fit test.[6]

We will then use the predicted probability of CRC and high-risk polyps from the selected best model to determine a cut-point above which a patient would be identified as at high risk for CRC and high-risk polyps. We will make an *a priori* plan to identify two risk stratification cut-points that improve sensitivity and specificity of current US Multi-Society Task Force on Colorectal Cancer guidelines, as previously described.[7] Defining sensitivity as the proportion of individuals with subsequent CRC or high-risk polyps who are classified as high risk at baseline, we will target the first cut-point to improve the sensitivity of US Multi-Society Task Force guidelines by 10 percentage points. Defining specificity as the proportion of individuals without subsequent CRC or high-risk polyps who were classified as low risk at baseline, we will target the other cut-point to improve the specificity of US Multi-Society Task Force guidelines by 10 percentage points. The population sensitivity and specificity of US Multi-Society Task Force guidelines were estimated at 68 percent and 54 percent, the median sensitivity and specificity observed in published literature.[7-11]

Model Validation and Comparison of Estimated Clinical Benefit in Validation Set

Model validation will be conducted using the validation set. Model discrimination will be assessed by the AUC. Model calibration will be assessed by Hosmer-Lemeshow goodness-of-fit test as well as comparing the predicted risk and observed risk of CRC and high-risk polyps for 10 deciles of risk groups. Potential clinical benefit will be assessed in the validation data using the model coefficients and cut-points identified in the training set, by estimated sensitivity and specificity for CRC and high-risk polyps and estimated rates of over- and under-use of colonoscopy. Overuse of surveillance colonoscopy will be defined as the proportion of individuals classified as high risk at baseline who did not develop CRC or high-risk polyps on follow-up.[7] Underuse of surveillance colonoscopy will be defined as the proportion of individuals classified as low risk at baseline who did not develop CRC or high-risk polyps on follow-up.[7] Improvement in specificity and sensitivity using the predictive model on the validation set will be assessed by McNemar test, and improvement in overuse and underuse will be assessed by 95 percent confidence intervals. Clinical benefit of using the predictive model over current guidelines will also be assessed using net reclassification improvement.[11]

References

1. Friedman J, Hastie T, Tibshirani R. Regularization Paths for Generalized Linear Models via Coordinate Descent. J Stat Softw. 2010;33(1):1-22.
2. Park MY, Hastie T. L1-Regularization Path Algorithm for Generalized Linear Models. J R Stat Soc Series B Stat Methodol. 2007;69(4):659-77.
3. Volinsky CT, Madigan D, Raftery AE, et al. Bayesian Model Averaging in Proportional Hazard Models: Assessing the Risk of a Stroke. J R Stat Soc Ser C Appl Stat. 1977;46(4):433-48.
4. Steyerberg EW, Eijkemans MJ, Harrell FE, et al. Prognostic modelling with logistic regression analysis: a comparison of selection and estimation methods in small data sets. Stat Med. 2000;19(8):1059-79.
5. Wang D, Zhang W, Bakhai A. Comparison of Bayesian model averaging and stepwise methods for model selection in logistic regression. Stat Med. 2004;23(22):3451-67.
6. Martínez ME, Baron JA, Lieberman DA, et al. A pooled analysis of advanced colorectal neoplasia diagnoses after colonoscopic polypectomy. Gastroenterology. 2009;136(3):832-41.
7. Liu L, Messer K, Baron JA, et al. A prognostic model for advanced colorectal neoplasia recurrence. Cancer Causes Control. 2016;27(10):1175-85.
8. Pinsky PF, Schoen RE, Weissfeld JL, et al. The yield of surveillance colonoscopy by adenoma history and time to examination. Clin Gastroenterol Hepatol. 2009;7(1):86-92.
9. Chung SJ, Kim YS, Yang SY, et al. Five-year risk for advanced colorectal neoplasia after initial colonoscopy according to the baseline risk stratification: a prospective study in 2452 asymptomatic Koreans. Gut. 2011;60(11):1537-43.
10. Laiyemo AO, Murphy G, Albert PS, et al. Postpolypectomy colonoscopy surveillance guidelines: predictive accuracy for advanced adenoma at 4 years. Ann Intern Med. 2008;148(6):419-26.
11. Pencina MJ, D'Agostino RB, Sr., D'Agostino RB, Jr., et al. Evaluating the added predictive ability of a new marker: from area under the ROC curve to reclassification and beyond. Stat Med. 2008;27(2):157-72.
